# Detection of rare plasmid hosts using a targeted Hi-C approach

**DOI:** 10.1093/ismeco/ycae161

**Published:** 2025-03-09

**Authors:** Salvador Castañeda-Barba, Benjamin J Ridenhour, Eva M Top, Thibault Stalder

**Affiliations:** Department of Biological Sciences, University of Idaho, Moscow, ID 83844, United States; Bioinformatics and Computational Biology Graduate Program (BCB), University of Idaho, Moscow, ID 83844, United States; Institute for Interdisciplinary Data Sciences, University of Idaho, Moscow, ID 83844, United States; Bioinformatics and Computational Biology Graduate Program (BCB), University of Idaho, Moscow, ID 83844, United States; Institute for Interdisciplinary Data Sciences, University of Idaho, Moscow, ID 83844, United States; Institute for Modeling Collaboration and Innovation, University of Idaho, Moscow, ID 83844, United States; Department of Mathematics and Statistical Science, University of Idaho, Moscow, ID 83844, United States; Department of Biological Sciences, University of Idaho, Moscow, ID 83844, United States; Bioinformatics and Computational Biology Graduate Program (BCB), University of Idaho, Moscow, ID 83844, United States; Institute for Interdisciplinary Data Sciences, University of Idaho, Moscow, ID 83844, United States; Institute for Modeling Collaboration and Innovation, University of Idaho, Moscow, ID 83844, United States; Department of Biological Sciences, University of Idaho, Moscow, ID 83844, United States; Institute for Interdisciplinary Data Sciences, University of Idaho, Moscow, ID 83844, United States; Institute for Modeling Collaboration and Innovation, University of Idaho, Moscow, ID 83844, United States; Université de Limoges, INSERM, CHU Limoges, RESINFIT, U1092, F-87000, Limoges, France

**Keywords:** plasmids, antibiotic resistance, Hi-C, target enrichment, soil

## Abstract

Despite the significant role plasmids play in microbial evolution, there is limited knowledge of their ecology, evolution, and transfer in microbial communities. This is partly due to the limitations of current methods in associating a plasmid with its host in microbiomes. To address this knowledge gap, we developed and implemented a novel approach to identify rare plasmid hosts by combining Hi-C, a proximity ligation method, with enrichment for plasmid-specific DNA. We hereafter refer to this approach as Hi-C+. We applied Hi-C and Hi-C+ to soil microbial communities in which we mimicked increasingly rare transfer of an antimicrobial resistance plasmid from a donor to a recipient. This was achieved by inoculating agricultural soil with mixtures of known plasmid-containing and plasmid-free cells at different proportions. We demonstrated that Hi-C can link a plasmid to its host in soil when the relative abundance of that plasmid-host pair is as low as 0.001%. Hi-C+ further improved the detection limit of Hi-C 100-fold and allowed the identification of plasmid hosts at the genus level. As a culture-independent approach, Hi-C+ will significantly improve our understanding of the range and frequency of spread of antibiotic resistance and other genes that are introduced into soil and other microbiomes.

## Introduction

Plasmids are mobile genetic elements that replicate separately from the bacterial chromosome and can transfer by conjugation to other closely and distantly related bacteria [1, 2]. It is now evident that horizontal gene transfer mediated by plasmids plays a crucial role in bacterial adaptation to changing environments [3, 4]. An important example is the role of plasmids in spreading antibiotic resistance. Plasmids can carry genes that encode resistance to 10 or more antibiotics [5], including so-called “drugs of last resort” [6–9].

To understand the spread of antimicrobial resistance and the horizontal transfer of any trait between bacteria, it is critical not to limit the study of plasmid transmission to human pathogens alone. Many antibiotic resistance genes (ARG) of concern in human pathogens are also prevalent in bacteria from animal and environmental habitats [10]. From these settings, plasmids facilitate the spread of ARG to human pathogens [11–13]. Therefore, it is crucial that we determine the conditions that facilitate the spread and persistence of plasmids in their natural habitats [14]. One critical challenge is to identify the bacteria that can acquire and retain resistance plasmids in the environment, as they can facilitate both the long-term persistence of plasmids and their spread to other members of the microbiome [15–17].

Current methods are unfortunately limited in their ability to identify plasmid hosts in situ, leaving an important knowledge gap in the field of plasmid ecology. While cultivation-based approaches allow tracking of plasmids in situ [18, 19], these methods are limited to the culturable fraction of bacteria, known to be less than 1% in soil [20]. Marking a focal plasmid with a fluorescent protein can enable the tracking and identification of putative new transconjugants [21–24]. However, modification of the plasmid may change its transferability and persistence [25], and successful fluorescence can be strain- and environment-specific [26, 27]. One could also quantify the presence of the focal plasmid using qPCR [28–30], or use shotgun sequencing to assess the relative abundance of the plasmid. Unfortunately, neither of these methods would give us information on the identity of the bacteria that have acquired the plasmid. Altogether, the limited ability of these methods to identify the hosts of a given plasmid in microbial communities is hampering the tracking of plasmid-mediated ARG spread.

**Figure 1:**
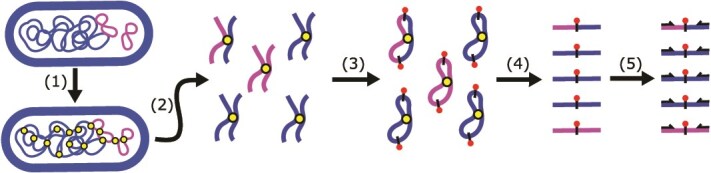
Hi-C method. (i) Before cell lysis, DNA is cross-linked with formaldehyde. Cross-links between DNA in proximity are depicted as yellow dots; (ii) cells are lysed, DNA is then purified and cut with a restriction enzyme; (iii) cut restriction sites are filled in using biotin-labeled nucleotides (red dots) and are then religated; (iv) DNA is sheared, and biotin-labeled fragments are enriched; (v) sequencing adapters are added, and Hi-C fragments are sequenced. The paired-end Hi-C reads are depicted as black arrows. Paired-end reads from chimeric fragments of DNA that linked the plasmid to its host can be used to identify that the pink plasmid was present in the blue bacteria.

The development of proximity ligation approaches, such as Hi-C and meta3C, has opened the door to further our insight into plasmid-mediated resistance spread in natural habitats. These approaches rely on crosslinking DNA within the cell, prior to cell lysis (Fig. 1). The physical contacts produced by the crosslinks provide quantitative and objective information on whether two segments of DNA share the same cellular compartment [31–33]. This information can help understand plasmid and phage-host interactions and aid in reconstructing the reservoirs of ARG and plasmids in wastewater as well as in human and animal gut microbiomes [34–40].

Despite its implementation for microbiome analysis, Hi-C suffers from low sensitivity in the detection of specific targets in a microbial community, just like most non-targeted metagenomic approaches. This is particularly problematic when monitoring plasmid spread, as transfer in natural habitats occurs at very low frequencies [41–43]. Furthermore, many resistance plasmids within microbial communities are present in members that are in low abundance [15, 17]. To overcome this challenge, we paired Hi-C with a target enrichment approach, hereafter referred to as Hi-C+. We first determined the detection limit of Hi-C for linking a specific plasmid to its host in soil. Subsequently, we showed that Hi-C+ can (i) improve this detection limit by enriching the Hi-C DNA library with DNA of a given plasmid, and (ii) facilitate genus-level identification of new plasmid hosts.

## Materials and methods

### Experimental design

**Figure 2:**
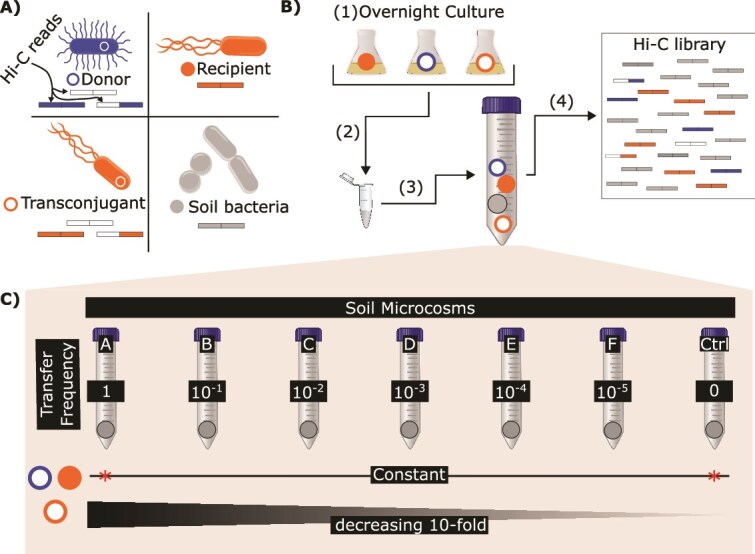
Overview of soil microcosm setup. (A) Legend for colors used to represent the donor, recipient, transconjugant, and soil bacteria. The types of Hi-C reads that can be generated by each bacterium are also depicted. (B) To accurately quantify the limits of Hi-C in detecting transconjugants, we set up soil microcosms to which we added a specific amount of donor, recipient, and transconjugant. An experimental flow chart of the soil microcosms is depicted: (i) donor, transconjugant, and recipient were grown overnight. (ii) Appropriate amount of each bacterium was pooled and (iii) subsequently added to soil. A visual representation of the composition of each microcosm is depicted in panel C. (iv) To limit opportunities for plasmid transfer in our experimental design, soil from each microcosm was immediately crossed-linked and underwent the Hi-C library preparation. Hi-C libraries were then sequenced. (C) Visualization of soil microcosm composition. The decreasing transconjugant frequency simulates scenarios where plasmid transfer is increasingly rare and allows us to measure the detection limit of Hi-C. *No recipients were added to soil microcosm A, ctrl soil microcosm contained recipient, a plasmid-free donor strain and no transconjugant.

To determine the detection limit of Hi-C, we designed an experiment that mimicked a scenario where a plasmid-carrying bacterial strain is introduced into soil, and subsequently transfers its plasmid to a soil bacterium (Fig. 2, Table 1). We refer to this initial plasmid-carrying strain as the donor, the bacteria that can acquire the plasmid as the recipients, and those to which the plasmid has transferred as the transconjugants. Rather than inoculating donors and recipients and letting plasmid transfer occur at unknown frequencies, we inoculated donors, recipients, and transconjugants at a series of known densities (Fig. 2B, Table 1). This allowed us to accurately assess the Hi-C method and determine its limit in detecting contacts between DNA from a known plasmid and the chromosomes of the transconjugants and donors.

**Table 1:** Soil microcosms imitating plasmid transfer scenarios with densities of each bacterial strain^1^ added and targeted “transfer frequencies”.

**Soil microcosm**	**Donor (D)**	**Recipient (R)**	**Transconjugant (T)**	**Soil community**	**Transfer frequency (T/R)**	**Hi-C**	**Hi-C +**
	(cfu/g)			(bacteria/g)^5^			
A	1.5 × 10^6^	0	1.7 × 10^8^	3.2 × 10^9^	1^*^	✓	4
B	0.9 × 10^6^	1.3 × 10^8^	1.2 × 10^7^	3.2 × 10^9^	10^−1^	✓	✓
C	1.5 × 10^6^	1.7 × 10^8^	1.4 × 10^6^	3.2 × 10^9^	10^−2^	✓	✓
D	1.9 × 10^6^	1.1 × 10^8^	1.9 × 10^5^	3.2 × 10^9^	10^−3^	✓	✓
E	1.2 × 10^6^	0.9 × 10^8^	1.4 × 10^4^	3.2 × 10^9^	10^−4^	✓	✓
F	0.9 × 10^6^	0.9 × 10^8^	1.3 × 10^3^	3.2 × 10^9^	10^−5^	3	✓
Control^2^	1.5 × 10^6^	0.9 × 10^8^	0	3.2 × 10^9^	0	✓	✓

^1^Donor: *E. coli* K12 MG1655Nal::gfp (pB10), Recipient: *Pseudomonas putida* KT2442, Transconjugant: *P. putida* KT2442 (pB10).

^2^Control microcosm is soil inoculated with plasmid-free *E. coli* K12 MG1655 Nal::gfp and Recipient.

^3^Hi-C library for Soil Microcosm F was not sequenced. Preliminary results indicated this would be beyond the detection limit.

^4^Hi-C+ library for Soil Microcosm A was prepared, but not sequenced. Preliminary results indicated this would work well and we opted to leave it out in exchange for higher sequencing depth on the other Hi-C+ libraries.

^5^Soil bacteria were estimated using 16S rRNA quantification, see methods for more details. See Table S1 for representation of the cell densities of D, R and T as relative abundances, relative to the soil bacterial densities.

^*^The scenario in microcosm A mimics a case where all the recipients acquired the plasmid, making the transfer frequency 1.

✓Hi-C and Hi-C+ libraries were prepared and sequenced.

### Strains and soil

Soil was obtained from the USDA ARS Northwest Irrigation and Soils Research Laboratory in Kimberly, ID and stored at 4°C until the time of the experiment. The three strains used in our study were the plasmid donors *Escherichia coli* K-12 MG1655Nal::gfp, containing plasmid pB10::rfp, hereafter briefly named pB10 [44], the plasmid-free recipient *Pseudomonas putida* KT2442, and the transconjugant *P. putida* KT2442 (pB10) [45, 46]. The original strains and their sequenced genomes were obtained from previous studies [44–46]. The sequence for *E. coli* K-12 MG1655 was manually modified to include the mini-*Tn*5-*gfp* transposon.

All three strains were grown overnight in lysogeny broth (LB; Thermo Fisher Scientific, Waltham, MA, USA) with shaking at 200 r.p.m. The donor was grown at 37°C in broth containing nalidixic acid (50 mg. L^−1^) and kanamycin (50 mg. L^−1^). The recipient was grown at 30°C in broth containing rifampicin (100 mg. L^−1^), and the transconjugant at 30°C in broth containing rifampicin (100 mg. L^−1^) and kanamycin (50 mg. L^−1^).

### Set up of soil microcosms

We added mixtures of donors, recipients, and transconjugants to 6 grams of soil in 50-ml Falcon tubes (see Table 1). To determine the number of bacteria to be added to each soil microcosm, we first estimated the total number of soil bacteria by quantifying the copy number of the 16S rRNA gene by qPCR. This protocol was followed as described by Hill *et al* [47], using the primers designed by Liu *et al* [48] that target the region in V3-V4 of the 16S rRNA gene*.* The sequences for the forward primer, reverse primer, and probe are 5′-CCTACGGGDGGCWGCA-3′, 5′-GGACTACHVGGGTMTCTAATC-3′ and (6FAM) 5′-CAGCAGCCGCGGTA-3′ (MGBNFQ). We used the 16S rRNA copies to estimate the bacteria per g of soil, assuming an average of 4.2 copies per bacterial chromosome [49]. Using this value, 5.28 × 10^8^ bacteria/g of soil, we estimated the number of donors, recipients, and transconjugants to be added to each soil microcosm to obtain the relative abundances shown in Table S1. Densities of donor, recipient, and transconjugant in the overnight cultures were estimated through plating on selective media and used these to determine the volume of cell suspensions added to each soil microcosm.

After overnight growth, the cultures were centrifuged for 3 min at 5000 × *g* and pellets were resuspended in 1/10^th^ volume of phosphate-buffered saline pH 7.4 (PBS) (Thermo Fisher Scientific, Waltham, MA, USA). For each soil microcosm (Table S1) the appropriate volume of each cell suspension was added into a tube and supplemented with PBS to a total volume of 150 μl. In a 50 ml falcon tube, 6 *g* of soil was mixed with this 150 μl mixture of donors, recipients, and transconjugants. The soil was thoroughly mixed with a sterile spatula and we immediately proceeded with preparation of Hi-C libraries.

### Sample processing and Hi-C library preparation

The bacterial fraction was extracted and purified from each soil microcosm by first resuspending the soil in 27 ml of sterile 2% sodium hexametaphosphate (Sigma-Aldrich, St. Louis, MO, USA), followed by vortexing for 5 min, then serially diluting and plating on selective media to obtain the plate count data (Table 1 and Fig. 2). The vortexed samples then underwent centrifugation at 150 × *g* for 30 sec. The supernatants were recovered into 50 ml falcon tubes and centrifuged at 10 000 × *g* for 10 min. The resulting pellets were resuspended each in 6 ml of Tris-Buffered Saline (TBS) (Thermo Fisher Scientific, Waltham, MA, USA), placed on a 5 ml OptiPrep™ cushion (Sigma-Aldrich, St. Louis, MO, USA), and centrifuged for 20 min at 10 000 × *g*. The top layer from the density gradient was recovered and centrifuged for 10 min at 17 000 × *g*. The pellets were resuspended in TBS and aliquoted into five micro-centrifuge tubes. Each aliquot was centrifuged for 5 min at 17000 × *g*, the pellet was resuspended in 1 ml of cross-linking solution (1% formaldehyde [Thermo Fisher Scientific, Waltham, MA, USA]) and incubated for 20 min at room temperature. Then, 100 μl of quenching solution (125 mM glycine (Sigma-Aldrich, St. Louis, MO, USA)) was added to each sample, followed by incubation for 20 more minutes. Samples were centrifuged at 17 000 × *g* for 5 min and the supernatant was discarded. The pellet was washed with 1 ml of TBS, centrifuged at 17 000 × *g* for 5 min, and stored at −20°C until library preparation. The cross-linked samples were sent to Phase Genomics^©^ for preparation of Hi-C libraries using their ProxiMeta™ kit according to the associated protocol.

### Preparation of Hi-C+ libraries

In this study we developed the combination of Hi-C with target capture and named it Hi-C+. This method consists of target DNA enrichment within the Hi-C libraries using the myBaits^®^ (Daicel Arbor Biosciences, Ann Arbor, MI, USA) custom target enrichment protocol. Design of probes was carried out by Daicel Arbor Biosciences, with 70mer probe length and 20 bp tiling over the entire length of the genome sequence of plasmid pB10. Probes were screened against the *E. coli* K-12 MG1655 and *P. putida* KT2442 reference sequences using Daicel Arbor Biosciences proprietary software. Those with high similarity to the references were removed, along with probes that had a significant percent of simple repeats or low-complexity DNA (>50% soft-repeat-masking, screened by Daicel Arbor Biosciences using RepeatMasker). Of the 3253 candidate probes, 4 were removed for having high similarity to the reference (between 92 and 100%).

Hi-C libraries were first pre-treated for compatibility with myBaits^®^ target enrichment kit. The depletion reaction was carried out using 200 ng of library DNA as input. Each library underwent four rounds of PCR amplification with KAPA HiFi HotStart ReadyMix (Roche Holding AG, Basel, CH) and universal primers for amplifying Nextera libraries. The amplification reactions were pelleted, resuspended in 30 μl of Dynabeads™ MyOne™ Streptavidin C1 beads (Invitrogen, Waltham, MA, USA), and pelleted on a magnetic particle concentrator. The supernatant was removed and cleaned using Zymo Research^©^ DNA Clean and Concentrator kit (Zymo Research^©^, Irivine, CA, USA). The resulting cleaned DNA was used as input for the target enrichment reaction.

The target capture reactions were carried out using the myBaits “v4” chemistry and associated v4.01 manual, with modifications for the high-sensitivity protocol. The temperature for hybridization and washing steps was changed to 62°C. In the first enrichment (step 1.2.2 in manual), we used 4.4 μl of baits and 1.1 μl of nuclease-free water. After the first enrichment, two amplifications per capture reaction were carried out (step 3.3 in the manual). The purified PCR products were combined, concentrated to 7 μl using Zymo Research^©^ DNA Clean and Concentrator kit, and used as input for a second round of target capture. For the second enrichment (step 1.2.2 in manual), we used 1.1 μl of baits and 4.4 μl of nuclease-free water. All other steps were carried out as instructed in the v4.01 manual.

### Sequencing of Hi-C and Hi-C+ libraries

Hi-C and Hi-C+ library size selection, quantification, and pooling were carried out by the IIDS Genomics and Bioinformatics Resources Core at the University of Idaho (Moscow, ID, USA). The libraries were then sequenced at the University of Oregon sequencing core (Eugene, OR, USA). The six Hi-C libraries were sequenced using two lanes of HiSeq 4000, 2 × 100 bp paired-end, while the six Hi-C+ libraries were sequenced on three lanes of NovaSeq 6000, 2 × 150 bp paired-end reads. The number of paired-end reads obtained from each library was as follows: A: 102818926, B: 97688195, C: 155702732, D: 127023749, E: 146798824, Ctrl: 103992690, B+: 67166513, C+: 466371775, D+: 56882152, E+: 83617103, F+: 405252012, Ctrl+: 58053294.

### Data analysis

The sequencing reads first underwent read trimming and filtering using fastp with default arguments and minimum length requirement of 50 base pairs [50]. We then aligned our data to our reference sequences using BWA Mem with the -5SPY options [51]. Hi-C and Hi-C+ read counts were subsequently analyzed using a custom python script. In this script, we binned together all the paired-end reads for which at least one end mapped to the sequence of pB10 and the other end mapped the sequence of *E. coli* MG1655, *P. putida* KT2442*,* or pB10. All scripts and detailed descriptions are available on GitHub (https://github.com/scastanedabarba/hic_targetcapture).

### Mathematical models

The model for predicting bacterial count was fitted using the glm.nb function in the MASS v7.3–58.2 R package. Our model is a negative binomial regression (Equation 1) with a response variable of bacterial count (cfu/g) and predictor variables Pputida-pB10 Hi-C reads and sequencing depth.

### De novo taxonomic classification of pB10 hosts

Trimmed reads were first aligned only to the *E. coli* MG1655 and pB10 sequences using BWA Mem with the -5SPY options [51]. After alignment, a custom python script was used to extract paired-end reads whereby one pair was aligned to pB10 and the other was unaligned. The same criteria defined for filtering alignments in our “data analysis” section were used here. The unaligned portion of these reads were output to new FASTA files and underwent taxonomic classification using Kraken2 with minimum confidence score of .1, using the standard database [52]. Taxonkit and csvtk were then used to convert taxids to full taxonomic lineages [53], followed by use of a python script to calculate metrics for each classification.

## Results

### Hi-C detects a plasmid-host pair present at a relative abundance of 10^−5^

We set up seven soil microcosms with various mock plasmid transfer scenarios (Table 1, Fig. 2A and C). Each microcosm represents a scenario where the plasmid has transferred to an increasingly smaller fraction of the recipients, resulting in a declining transfer frequency and overall proportion of transconjugants.

**Figure 3:**
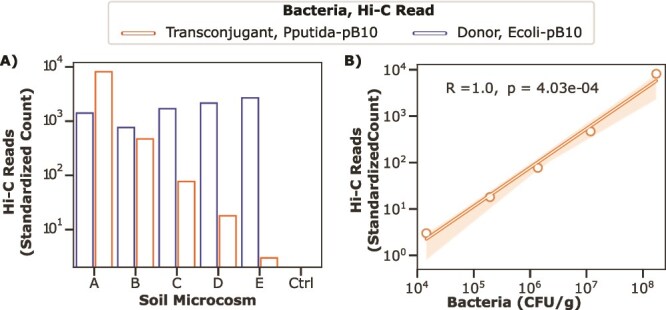
Detection of transconjugants and donors by Hi-C. (A) Reads linking the plasmid to the transconjugant and donor chromosome were detected in all soil microcosms tested, except for the control. To account for differences in sequencing depth, Hi-C read count was standardized to the lowest sequencing depth. (B) Relationship between transconjugant bacteria plated (Table 1) and Hi-C reads observed. A Pearson correlation coefficient was computed and is displayed in the top left.

Our first goal was to determine if the Hi-C method could detect Hi-C reads associating pB10 with the chromosomes of a decreasing number transconjugants and a constant number of donors in each of the soil microcosms. To do this, we generated and sequenced Hi-C libraries from soil microcosm A through E and control (Table 1). When analyzing the paired-end Hi-C reads, we specifically looked for those where one aligned perfectly to the *P. putida* KT2442 (transconjugant) or *E. coli* MG1655 (donor) reference sequences and the other to pB10. These reads are respectively denoted as Pputida-pB10 and Ecoli-pB10 Hi-C reads; this hyphenated nomenclature indicates the two genomes to which paired-end reads aligned. We detected Pputida-pB10 and Ecoli-pB10 Hi-C reads in all but the control Hi-C libraries (Fig. 3A). Importantly, transconjugants were still detected even when the donor was 100 times more abundant (E in Table 1 and Fig. 2). Additionally, there was a strong correlation between the amount of transconjugants added and observed Pputida-pB10 Hi-C reads (Fig. 3B). Our results demonstrate that Hi-C can distinguish a plasmid present in multiple hosts in a natural soil community, even when one host is present at a low relative abundance.

To monitor the spread of antimicrobial resistance, researchers should be able to enumerate new plasmid-host pairs in soil or other environments. To address this, we built a quantitative model that relates Hi-C reads to bacterial counts, using the data collected from the sequenced soil microcosms in conjunction with plate count data (Fig. 3A and Table 1). To account for the effect of sequencing depth, we first randomly subsampled the paired-end reads from each soil microcosm at the following sequencing depths; 50, 60, 70, 80, and 90 million paired-end reads. We then performed negative binomial regression (Equation 1) with a response variable of bacterial count (cfu/g) and predictor variables Pputida-pB10 Hi-C reads (*z-score* = 16 891, *P* < .001) and sequencing depth (*z-score* = −3092, *P* <. 001).

(1) Bacterial count = *e*^24.03^ * Pputida-pB10^0.83^ ÷ sequencing_depth^0.828^

Using this model will enable future estimations of the prevalence of an introduced plasmid in an indigenous bacterial community.

Additionally, we determined the detection limit of Hi-C by calculating the minimum number of plasmid hosts needed in a bacterial community to allow detection of at least one Hi-C read that links the focal plasmid to its host with high confidence. First, using Poisson probability we identified that a library needed to have a mean of 9 plasmid-host DNA fragments, for there to be a high probability [P(X > 0) = 99.99%] of detecting at least one plasmid-host Hi-C read within a sequencing run. Using equation 1, we calculated that given a sequencing depth of 90 million paired-end reads, this corresponds to 3.3 × 10^4^ bacteria/g of soil. Presented as a relative abundance of the number of bacteria in our soil microcosm, 3.2 × 10^9^ bacteria/g of soil, this equates to 10^−5^. The detection of transconjugant Hi-C reads in scenario E, which lies beyond the detection limit, shows that reads can still be detected below the identified limit, even though it is less likely. Notably, this detection limit is dependent on the desired number of observed Hi-C reads. Maintaining the same sequencing depth but increasing the desired observed Hi-C reads to 10 or 100 would respectively result in detection limits of 1.1 × 10^5^ and 7.1 × 10^5^ bacteria/g of soil. Altogether, our results demonstrate that when a plasmid-host pair is present in a community at a relative abundance of 10^−5^, we can expect to detect it in 90 million paired-end reads.

### Hi-C+, enriching a Hi-C library with plasmid deoxyribonucleic acid through target capture

**Figure 4:**
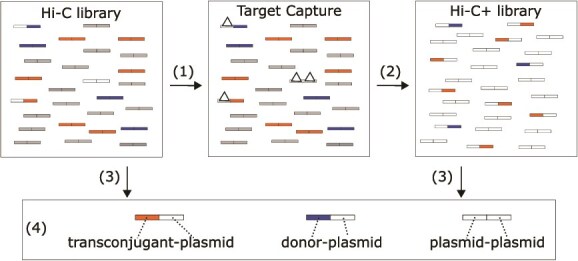
Overview of Hi-C+. (i) DNA from a Hi-C library was used as input for the target capture reaction. RNA baits complementary to the sequence of our plasmid (white triangles) were designed and applied to the Hi-C library. (ii) The bait-bound library was purified and PCR-amplified, generating Hi-C+ libraries enriched for plasmid-specific DNA. (iii) Hi-C and Hi-C+ libraries were sequenced. (iv) Data analysis of these libraries consists of looking for all plasmid-associated Hi-C reads.

In natural microbiomes plasmid transfer is often rare, such that transconjugant proportions can be below 10^−5^ [41–43], the detection limit of Hi-C. To circumvent this limitation, we set out to improve the detection limit of this method for studies that monitor one focal plasmid in a bacterial community. This was accomplished by combining Hi-C with a target enrichment approach, a method we refer to as Hi-C+ (Fig. 4). Hi-C+ was applied directly to DNA from the Hi-C libraries of soil microcosms B through F and the control microcosm, yielding so-called Hi-C+ libraries (see Table 1). To minimize experimental variation, all reactions were carried out in parallel.

**Figure 5:**
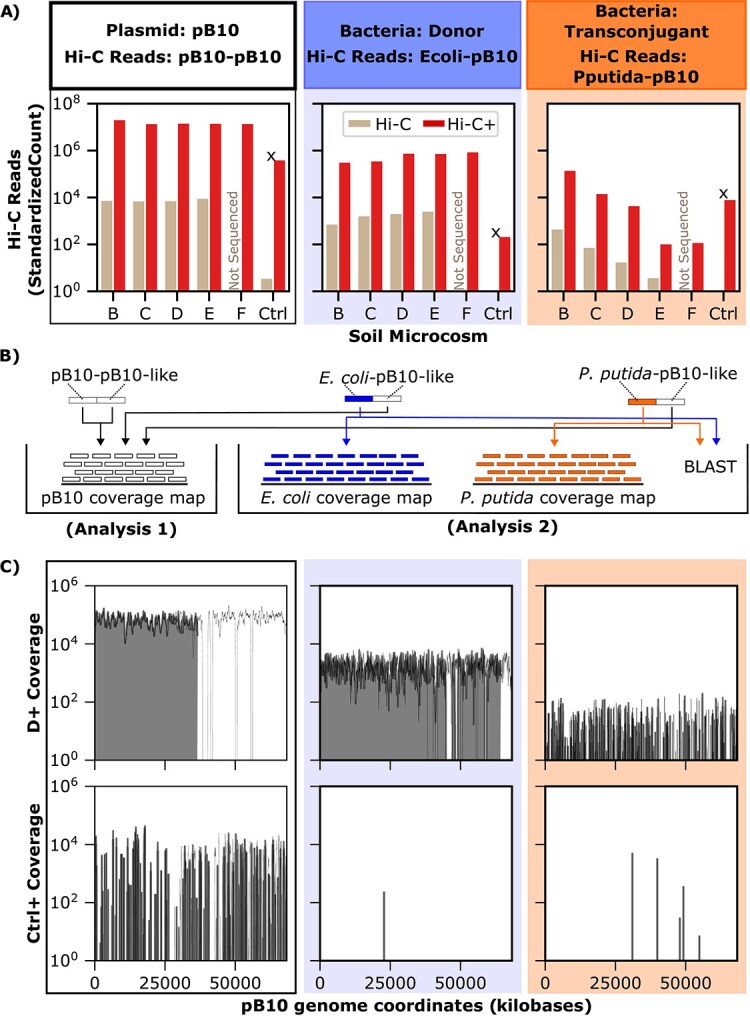
Detection of plasmid-linked reads in Hi-C+ libraries. (A) Detection of pB10-pB10, Ecoli-pB10, and Pputida-pB10 reads in each Hi-C and Hi-C+ library. We only retained alignments mapped to a single reference to prevent false enrichment signals from similar DNA on the plasmid and bacterium sequences. Probe design was carried out on reference genomes that did not contain the mini transposons with *gfp* in our marked *E. coli* MG1655 and *rfp* in pB10. As a result, some probes bind to both the pB10 and *E. coli* genomes. ^X^As shown by coverage plots in panel C, although the control microcosm had a high number of plasmid-associated reads, these only aligned to five short plasmid segments and likely correspond to non-specific enrichment of other similar DNA fragments in soil. (B) To investigate this further, we carried out an analysis on the plasmid-linked reads in the control soil microcosm. Arrows demonstrate the analyses carried out on each of the Hi-C+ reads. Analysis 1 investigates where the pB10 segment of plasmid-like reads align on the plasmid reference genome. Analysis 2 determines where the *P. putida* KT2442 and *E. coli* MG1655 segments of plasmid-like reads align on their respective reference genomes. This is accompanied by a BLAST search of the sequence against the NCBI nucleotide database to determine whether the region to which the reads align is conserved across bacteria. (C) Comparison of results from analysis 1 carried out on reads from a microcosm to which we added pB10 (D+) and the control (ctrl+). See Fig. S2 for all coverage maps. Gaps in coverage maps for D+ pB10-pB10 and Ecoli-pB10 Hi-C+ reads are areas of the pB10 genome that were identical to regions of the *P. putida* KT2442 or *E. coli* MG1655 genomes. Results from analysis (2) are shown in Fig. S3. Each column is color-coded to indicate the Hi-C reads for which data is presented.

Comparison of the number of plasmid-associated reads (pB10-pB10, Ecoli-pB10 and Pputida-pB10) show that Hi-C+ enrichment was successful (Fig. 5A). Compared to Hi-C, the Hi-C+ libraries contained on average 3573 (SE ± 780) times more pB10-pB10 reads, 572 (SE ± 138) times more Ecoli-pB10 reads, and 343 (SE ± 186) times more Pputida-pB10 reads. A higher pB10-pB10 enrichment is due to (i) the close contact of plasmid-plasmid DNA, increasing the frequency of pB10-pB10 Hi-C reads, and (ii) the fact that baits can bind both sides of those Hi-C reads. This result demonstrates that Hi-C+ can increase the proportion of plasmid DNA in Hi-C libraries.

### Target capture enriched genes commonly found in a soil microbiome

While Hi-C+ enriched pB10 plasmid DNA in the Hi-C libraries from soil microcosms B-F, we also observed enrichment in the control soil microcosm that was not inoculated with pB10 (Figs 5 and S1). This result suggests that members of the soil community contain DNA fragments identical to that of our plasmid and donor and recipient strains. Indeed, IncP-1 plasmids and some pB10 genes such as transposons, integrons and resistance genes are ubiquitous in soil [15, 54, 55]. Another possibility is that that library from the control soil sample had been cross-contaminated. While a sample negative control would have allowed us to rule out this hypothesis, the observed coverage pattern indicates non-specific enrichment. Contamination would likely manifest as a more homogeneous coverage across pB10 with low read depth. In contrast, we observed high read depth concentrated around specific genes, which are likely found in other bacteria. Therefore, we hypothesized that in absence of pB10, target capture enriched sequences that are non-specific to our focal plasmid but commonly found in the soil bacterial community. We refer to these sequences as pB10-like in Fig. 5, to differentiate them from those observed in microcosms to which we added our focal plasmid.

Because the positive Hi-C+ signal in the negative control soil microcosm could interfere with tracking a focal plasmid, we explored these results in more detail. First, we looked at where the pB10-like reads aligned on the pB10 reference genome (Fig. 5B). Reads mapping to pB10 aligned to common genes among plasmids. For example, genes from the *trb* and *tra* operons (see Fig. S2 for all genes detected). Additionally, coverage provided by pB10-like reads in the control microcosm was sparser than for a microcosm to which we added pB10 (Fig. 5C). The difference in coverage and detection of genes commonly found in soil indicates that those pB10-like reads are likely a reflection of the existing reservoir of mobile genetic elements in soil. Next, we examined the chromosomal side of Ecoli-pB10-like and Pputida-pB10-like Hi-C reads (Fig. 5B). A BLAST search of the few *E. coli* and *P. putida* segments revealed that these were present across many bacteria within their respective genus (Fig. S3). This further reinforces the notion that the detection of plasmid-associated reads in our negative control is due to the presence of DNA identical to our inoculated bacteria and plasmid in soil. In sum, despite some background enrichment by Hi-C+, it is possible to distinguish noise from focal plasmid-host links.

### Exploitation of plasmid coverage and uniqueness helps discriminate specific Hi-C+ reads from non-specific target

The detection of pB10-like reads in our control soil microcosm indicates that additional steps are needed to discriminate pB10-specific from non-specific enriched sequences. We therefore incorporate two key metrics: plasmid coverage and unique plasmid bases. First, if a target capture reaction specifically enriches a Hi-C library with our focal plasmid pB10, then we expect that those Hi-C+ reads map over the entire length of the plasmid sequence. This would result in a higher and more even plasmid coverage than a non-specific enrichment reaction (see Fig. 5C). Second, the inclusion of a control microcosm enabled us to identify the segments unique to pB10. At each location in the pB10 sequence, we compared whether that base was detected in sequencing data for the control microcosm. Locations that were not detected in the control microcosm, and were thus likely specific to our focal plasmid, were denoted “unique plasmid bases”. We then calculated the percentage of these unique plasmid bases. Similarly to plasmid coverage, we expect that successful enrichment results in a higher percentage of unique plasmid bases detected in the treated soils. Using this percentage in combination with the plasmid coverage can facilitate differentiation between specific and nonspecific target-capture reactions.

**Figure 6:**
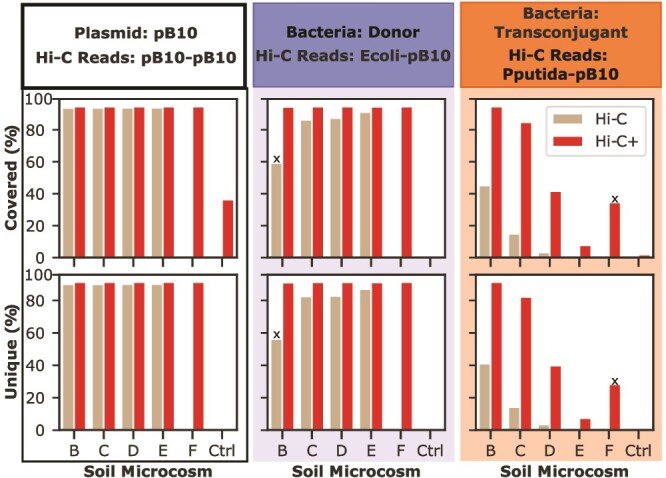
Plasmid coverage and unique bases help identify specific target capture reactions. Specific enrichment of a focal plasmid in the treated microcosms can be discerned by comparing the percentage of plasmid coverage (Top) and unique plasmid bases captured (bottom) to the control microcosm. Each column is color-coded to indicate the Hi-C and Hi-C+ reads for which data is presented. ^X^Two deviations from our expectations were observed in our results. The first is the lower score for Hi-C donor reads in microcosm B. We expect this is a result of there being a higher amount of transconjugant than donors in this microcosm, which affected their proportions in the sequenced library. The second is the higher scores for transconjugant Hi-C+ reads in microcosm F compared to E. We expect this occurred due to differences in the extraction efficiency of the bacterial portion from soil (see methods) coupled with experimental variation in target capture.

We applied our plasmid coverage metrics to the paired-end Hi-C reads from each soil microcosm. Consistent with our expectations, pB10-pB10 Hi-C reads and those indicating the presence of the donors (Ecoli-pB10) had a high plasmid coverage and percentage of unique plasmid bases detected in every pB10-treated soil microcosm (Fig. 6). This was the case for Hi-C alone and Hi-C+. For reads indicating the presence of transconjugants (Pputida-pB10), the Hi-C+ libraries from microcosms B, C, D, and F showed high plasmid coverage and percentage of unique plasmid bases (Fig. 6). Most importantly, in the control, the coverage was much lower than the microcosms to which we added pB10 (Fig. 6). This highlights that focal plasmid coverage, especially intra-plasmid Hi-C reads, can be used to assess whether a plasmid of interest was successfully enriched for.

### Hi-C+ can identify the new hosts of a focal plasmid in soil

**Figure 7:**
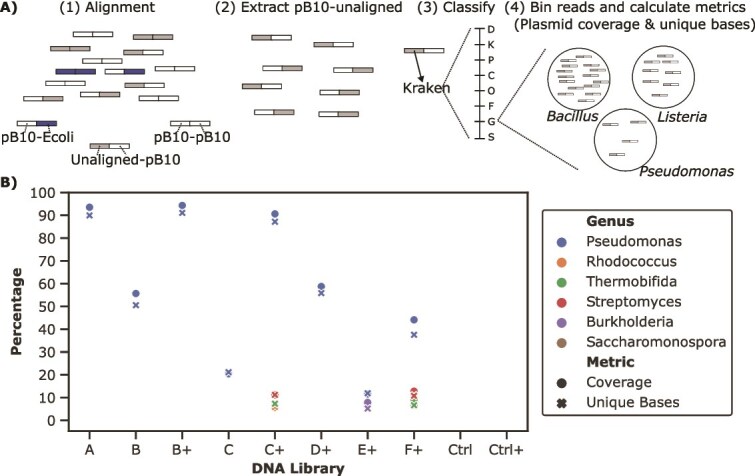
De novo identification of pB10 hosts. (A) Overview of data processing pipeline. (i) reads are first aligned only to pB10::rfp and *E. coli* MG1655 reference sequences, generating the plasmid-associated Hi-C reads depicted at the bottom.(ii) pB10-unaligned reads are extracted. (iii) The unaligned portion of pB10-unaligned reads is used to assign taxonomic classification using Kraken2. Classification is assigned at each taxonomic rank, from domain (D) to species (S). (iv) Read binning and calculation of genome coverage metrics (plasmid coverage and percent unique plasmid bases). An example of read binning at the genus level is shown. Reads are binned within each classification, here *Bacillus*, *Listeria* or *Pseudomonas,* and the genome coverage metrics are calculated for each bin. (B) Genus level identification of pB10 hosts and associated metrics. The plus signs in the X axis differentiate results from standalone Hi-C libraries, and the Hi-C+ libraries to which we applied target capture. Genera with less than 5% plasmid coverage and unique plasmids bases were excluded from the visualization. Except for the controls, the libraries with no genus above the established cutoff were also excluded from visualization.

In our experimental design, we knew the identity of the transconjugant, *P. putida* KT244 (pB10) and had its reference sequence, which facilitated its detection in each of our soil microcosms. However, researchers seeking to use Hi-C+ to monitor the transfer of a plasmid from a donor strain to the members of any microbiome will have to use taxonomic classifiers to identify the putative new hosts of their focal plasmid. We sought to emulate this process by attempting a de novo identification of our *P. putida* KT2442 transconjugant. An overview of the data processing pipeline is shown in Fig. 7A. Only reads that consisted of pB10 sequence on one side and unknown sequence on the other, designated “pB10-unaligned”, were retained. To avoid potential false positive Hi-C reads coming from the donor, reads aligned to the donor genome were excluded from downstream analysis. For the assignment of taxonomic classification, the unaligned portion of pB10-unaligned reads were used as input for Kraken2 [52]. For each taxonomic rank, classified reads were used to calculate the plasmid coverage metrics established in the previous section (plasmid coverage and percentage of unique plasmid bases detected) (Fig. 7A).

We applied this workflow to the Hi-C and Hi-C+ reads from each of our soil microcosms. In libraries A, B+ and C+, we were able to identify *Pseudomonas* as the transconjugant with high confidence. That is, reads linking pB10 to this genus had high scores for plasmid coverage and unique plasmid bases detected (Fig. 7B). The *Pseudomonas* genus was also identified as the transconjugant in libraries B, C, D+, and F+, although with lower percentage of plasmid coverage and unique plasmid bases detected. Notably, below 20%, we identified numerous other genera as transconjugants. These hosts are likely false positives that result from bacteria in soil that carry plasmids similar to our focal plasmid. Identification with high confidence below the genus level is difficult to attain due to the short sequence (less than 200 bp) and the high similarity between the genomes of species within a genus. While sequences could be classified at the species level, these would be a small portion of reads, which would result in erroneous identification of putative hosts at the species level.

## Discussion

Plasmids are important mediators of horizontal gene transfer in microbial communities [56]. Across multiple habitats they play a crucial role in mobilizing ARG, which can eventually transfer to human pathogens [14]. It is therefore pivotal to detect plasmid transfer and identify new plasmid hosts in natural communities and complex matrices. To this end, we developed Hi-C+, a method that can identify rare plasmid hosts. The implementation of Hi-C+ can provide important insights into the key players and factors that drive the vertical and horizontal transfer of ARG, which can in turn be used to design strategies to limit their spread.

We show that the standalone Hi-C method can be used to link a focal plasmid to its hosts in a diverse microbiome such as an agricultural soil. While Hi-C has previously been used to identify plasmid hosts [34–37, 57], a quantitative assessment of its detection limit had not been carried out. Here, we determine that plasmid hosts can be identified even when present at densities as low as 10^−5^ or 0.001% of the total bacterial community. Moreover, our findings suggest that Hi-C can discriminate rare transconjugants in the presence of the donors and the natural soil community and can even help identify the host taxon when combined with a target enrichment approach. Additionally, we defined a model that can be used to relate Hi-C reads to bacterial counts in cfu/g. Although this equation can be extrapolated to other plasmids, it is important to consider that plasmid size may influence the frequency of both intra- and inter-plasmid links as well as plasmid-chromosome links. However, we expect this effect to be minimal compared to the changes in the frequency of both the plasmid and its host, which can vary over several orders of magnitude. While the quantitative aspect of this method is limited to plasmid hosts present above a relative abundance of 10^−5^ and Hi-C datasets prior to application of target capture, it provides a way to obtain quantitative information on plasmid containing cells. Such information can be useful for understanding the ecology of plasmids in natural habitats.

Our Hi-C+ approach increased the detection limit of the Hi-C method for associating a plasmid with its hosts in soil by using target enrichment. The degree of enrichment varied between targets, with the plasmid donors being enriched on average 500-fold and the transconjugants 300-fold. These findings are comparable with those from previous studies employing target capture [58–60]. We observed a difference in degree of transconjugant enrichment across microcosms, with it decreasing as the number of added transconjugants decreased. We expect that this difference is due to the higher abundance of donors in most of our soil microcosms. In our target capture reactions, donor and transconjugant Hi-C reads are in competition for plasmid baits. The higher abundance of donor reads in most of the soil microcosm Hi-C libraries likely resulted in their more successful enrichment, which is further exacerbated by the use of multiple PCR cycles in the protocol. This highlights that, when using Hi-C+ for studying transfer of a focal plasmid, the original plasmid hosts (donors) need to drop in abundance below that of transconjugants to limit the extent to which they sequester the plasmid baits in the reaction. In sum, the combination of target capture with Hi-C enabled us to improve the detection limit of the method for linking a plasmid to its host.

We also showed that Hi-C+ can be used to identify the host of a focal plasmid in soil at the genus level. When attempting de novo identification of the transconjugant, we were able to do so at the genus level (*Pseudomonas* sp.) when the relative abundance of this strain in the soil community was as low as at 10^−7^. This limit corresponds to soil microcosm F, where reads linking pB10 to the *Pseudomonas* genus had >30% plasmid coverage and unique plasmid bases detected. Importantly, this represents a significant improvement compared to Hi-C alone. Similar confidence in host identification with Hi-C was only achieved in soil microcosm A, B and C, where the transconjugants were respectively present at 10^−1^, 10^−2^, and 10^−3^ of the total bacterial community. An important consideration here is the identification of a lower threshold for plasmid coverage that is used to confidently identify putative hosts. This threshold will vary according to the environment, type of focal plasmid, and sequencing depth. We therefore recommend that it be interpreted on an experiment-by-experiment basis and on a gradient. Putative hosts that have high coverage can be more confidently interpreted to carry the introduced plasmid, and this decreases as the coverage decreases. Comparisons to existing literature regarding plasmid host range should be considered when determining whether putative hosts that have low coverage can feasibly acquire a focal plasmid. Application of Hi-C+ combined with careful interpretation of identified hosts will significantly improve our ability to identify new transconjugants.

Lastly, we identified key limitations that need to be considered in future microbiome studies that use target capture on Hi-C reads. To limit Hi-C+ non-specific enrichment, a possible approach is the exclusion of regions commonly shared between plasmids in the probe design, e.g., accessory genes such as those encoding resistance, transposases, or conjugative transfer. One could also exclude areas of the focal plasmid that are common in the environment under study, such as regions common to IncP plasmids typically present in soil [55, 61, 62]. Nevertheless, we have proposed two plasmid coverage metrics to increase confidence in putative hosts identified by Hi-C+. We demonstrate that usage of plasmid coverage and unique plasmid bases detected increases confidence regarding the identification of plasmid hosts.

Altogether, we demonstrate the application of Hi-C and Hi-C+ in soil microcosms to detect and identify the host of a focal plasmid. We show that this approach can identify the host of a plasmid even when present in low abundance in a community, and thereby provide new insights into rare plasmid reservoirs in diverse habitats. Despite the fact that many microbes occur at low frequencies within their community, the rare biosphere remains an unexplored and often overlooked part of microbiomes [63]. These rare microbes may have critical roles in the ecology of microbial communities. Therefore, methods that can access this proportion of the microbiome are pivotal for unraveling the complex ecology of plasmids.

## Supplementary Material

Supplementary_Materials_ycae161

## Data Availability

All sequencing data pertaining to this project have been made available at the National Center for Biotechnology Information Sequencing Read Archive (SRP471772). All scripts used for data analysis are available on GitHub (https://github.com/scastanedabarba/hic_targetcapture).
